# Small Extracellular Vesicles Released from miR-211-5p-Overexpressed Bone Marrow Mesenchymal Stem Cells Ameliorate Spinal Cord Injuries in Rats

**DOI:** 10.1523/ENEURO.0361-23.2023

**Published:** 2024-02-09

**Authors:** Xianxiang Wang, Lei Ye, Ke Zhang, Lu Gao, Jin Xiao, Yiquan Zhang

**Affiliations:** Department of Neurosurgery, The First Affiliated Hospital of Anhui Medical University, Hefei, Anhui 230022, China

**Keywords:** bone mesenchymal stem cell, extracellular vesicle, miR-211-5p, Small extracellular vesicles z, spinal cord injury

## Abstract

Spinal cord injury (SCI) has become one of the common and serious diseases affecting patients’ motor functions. The small extracellular vesicles secreted by bone marrow mesenchymal stem cells (BMSCs) have shown a promising prospect for the treatment of neurological diseases. BMSCs were collected from rat bones. Osteogenic and adipogenic differentiation of BMSCs was further determined. Small extracellular vesicles were obtained by high-speed centrifugation. Dual-luciferase reporter assay was performed to demonstrate the targeting of miR-211-5p to the cyclooxygenase 2 (COX2) mRNA. qRT-PCR and Western blot assay were used for the detection of the mRNA and protein expression. ELISA was performed to estimate the levels of proinflammatory factors in spinal cord tissues. Our results showed that miR-211-5p targeted COX2 mRNA and regulated the protein expression of COX2 in BMSCs. Extracellular vesicles released from miR-211-5p-overexpressed BMSCs ameliorated SCI-induced motor dysfunction and motor evoked potential impairments. Extracellular vesicles released from miR-211-5p-overexpressed BMSCs ameliorated SCI-induced COX2 expression and related inflammatory responses. In conclusion, small extracellular vesicles released from miR-211-5p-overexpressed BMSCs ameliorate spinal cord injuries in rats.

## Significance Statement

The current study demonstrates that small extracellular vesicles released from miR-211-5p-overexpressed BMSCs ameliorate spinal cord injuries in rats.

## Introduction

As one of the most serious traumas in the central nervous system, spinal cord injury (SCI) is a serious complication of spinal fractures (Eckert and Martin, 2017). SCI is a central nervous system disease with high incidence, high mortality, high disability, and high cost, which has brought a heavy burden to patients and society (Fan et al., 2018). SCI can be divided into primary injury and secondary injury (Kjell and Olson, 2016). The primary injury is determined by the torsional force, compression force, and nerve transection experienced at the time of the injury (Chay and Kirshblum, 2020). Secondary damages such as blood spinal cord barrier disorder, ischemic edema, inflammation, lipid peroxidation, free radical generation, ion pathway damage, axon demyelination, nerve cell apoptosis, and scar formation can further aggravate the condition of SCI (Karsy and Hawryluk, 2019). Therefore, combating the secondary damage that occurs in the cascade, stimulating axon regeneration, and preventing spontaneous and continuous neuronal degeneration are feasible methods for the treatment of SCI (Fakhoury, 2015).

MicroRNA (miRNA) is a small noncoding RNA molecule that regulates mRNA degradation (Lu et al., 2005; Liu et al., 2014). The neuroprotective effects of miR-211-5p have been widely reported. It has been shown that miR-211-5p decreased the expression of cyclooxygenase 2 (COX2) and thus reduced focal brain ischemia-reperfusion injury in a rat model (Peng et al., 2020). It has also been proved to ameliorate the nerve and glial cell damage and apoptosis in an in vitro model of SCI (Zhang et al., 2021). In addition, previous studies have revealed that miR-211-5p in the hippocampus had an important regulatory effect on the regeneration of glial cells and the occurrence of depression in the rats (Tian et al., 2019).

Extracellular vehicles (EVs) are small vesicles that contain a series of RNA and proteins (Zhang and Yu, 2019). Multiple types of cells can produce EVs under different conditions (Koritzinsky et al., 2017). EVs can regulate the biological activity of recipient cells through the proteins, nucleic acids, or lipids they carry (Batrakova and Kim, 2015). In this research, bone marrow mesenchymal stem cells (BMSCs) were employed to achieve the overexpression of miR-211-5p, and the potential of EVs secreted from these BMSCs against SCI were examined.

## Materials and Methods

### Animals

Male Wistar rats were purchased from Shanghai Model Organisms. Ethical approval for the research protocol was obtained from the Ethics Committee of the First Affiliated Hospital of Anhui Medical University. Throughout the breeding period, all rats were provided with unrestricted access to food and water. The study employed a total of 32 rats, with eight rats allocated to each experimental group.

### BMSCs isolation

Four-week-old Wistar rats’ hindlimbs were stripped of the skin and muscle tissue attached to it to expose the rat's femur. The metaphyses at both ends of the femur and tibia were clipped to reveal the bone marrow cavity. DMEM/F12 medium (containing 10% fetal bovine serum and 1% double antibody) was used to flush out the cells in the medullary cavity of the bone. The marrow cavity cells were collected by centrifugation and planted into culture flasks and placed in an incubator for culture (37°C, 5% CO_2_ saturated humidity). Flow cytometry was used to select and identify BMSCs at passages 3–5. CD29, CD105, CD34, and CD45 were makers. Cells negatively expressed CD34 and CD45 and positively expressed CD29 and CD 105 were regarded as BMSCs.

### MicroRNAs

The miR-211-5p and relative control miRNA (NC) used in this study was synthesized by RiboBio Biotechnology as follows:

miR-211-5p: 5′-UUCCCUUUGUCAUCCUUUGCCU-3′;

5′-AGGCAAAGGAUGACAAAGGGAA-3′.

### Collection of EVs from BMSCs

Lipofectamine 2000 was used for the transfection of miRNAs to BMSCs at 3–5 passages for 48 h. The fetal bovine serum was centrifuged at 1,00,000 × *g* ultra-high speed, and the bottom sediment was discarded to obtain serum without EVs. Phosphate-buffered saline (PBS) was used to wash the BMSCs three times, and the complete medium containing the fetal bovine serum was replaced to culture the cells. After 36 h of culture, the cell supernatant was collected. The cell supernatant was centrifuged at 8,000 r/min for half an hour at 4°C to remove dead cells and cell debris. A 0.22 µm filter was used to filter out smaller cell debris. The obtained supernatant was sucked into the ultrafiltration tube and centrifuged at 6,000 × *g* for half an hour at 4°C to concentrate the supernatant to ∼200 μl. qEV kit was used to purify EVs according to the operating instructions. Electron microscopy was used for the detection of EVs.

### Dual-luciferase reporter assay

Luciferase Reporter Assay Substrate Kit - Firefly (ab228530; Abcam) was used for the dual-luciferase reporter assay in this study following the operating instructions.

### Osteogenic and adipogenic differentiation of BMSCs

Invitrogen-Mesenchymal Stem Cell Differentiation Medium (Thermo Fisher Scientific) was used to induce osteogenic differentiation. Invitrogen-Mesenchymal Stem Cell Differentiation Medium (Thermo Fisher Scientific) was used to induce adipogenic differentiation. Alizarin red S and oil red O are used to stain mesenchymal stem cells after osteogenic differentiation and adipogenic differentiation.

### Quantitative real-time PCR

qRT-PCR analysis was performed using a FastKing One Step RT-qPCR Kit (SYBR Green) purchased from Tiangen Biotech following the directions. β-Actin was used as an internal reference. The primers were as follows:

miR-211-5p: F: 5′-TTCCCTTTGTCATCCTTTG-3′,

R: 5′-GCAGGGTCCGAGGTATTC-3′;

COX2: F: 5′-CCATGTCAAAACCGTGGTGAATG-3′,

R: 5′-ATGGGAGTTGGGCAGTCATCAG-3′);

U6: F: 5′-CCTGCTTCGGCAGCACA-3′,

R: 5′-AACGCTTCACGAATTTGCGT-3′.

β-Actin F: 5′-GGAGATTACTGCCCTGGCTCCTAGC-3′,

R: 5′-GGCCGGACTCATCGTACTCCTGCTT-3′.

### Western blotting assay

Cell lysis buffer (Thermo Fisher Scientific) was used for the extraction of proteins. The protein of each mouse was separated and transferred to a PVDF membrane. The primary antibody with working solution including anti-CD9 (1:1,000), anti-CD63 (1:1,000), anti-Alix (1:1,000), anti-COX2 (1:1,000), and anti-GAPDH (1:1,000) were incubated with membranes at 6°C for 12 h.

### Enzyme-linked immunosorbent assay (ELISA)

ELISA was performed to evaluate the expression of interleukin (IL)-6, IL-1β, and tumor necrosis factor (TNF)-α in the spinal cords of rats by Rat IL-6 ELISA Kit (#ERA31RBX5, Thermo Fisher Scientific), Rat IL-6 ELISA Kit (#ERA31RBX5, Thermo Fisher Scientific), Rat IL-1 beta Uncoated ELISA Kit with Plates (#88-7013-22, Thermo Fisher Scientific) and Invitrogen TNF alpha Rat ELISA Kit (#BMS622, Thermo Fisher Scientific) following the instructions.

### The establishment of SCI model

In this study, we used spinal cord hemisection to establish a SCI model. A 3.5 vol% isoflurane was used to induce anesthesia in rats. The rat's back was depilated and thoroughly disinfected with Aner Iodine. A longitudinal incision along the midline of the back was made ∼2 cm centered on the T10 spinous process of the thoracic vertebra to expose the T10 spinous process and lamina. T9–T10 lamina was opened to reveal the white spinal cord and the posterior median vein of the spinal cord. The ophthalmic iris knife was inserted vertically from the posterior median vein and cut the right spinal cord to remove residual fibrous tissue. After the rat woke up, the right hindlimb could be seen paralyzed, and the left hindlimb was normal or slightly restricted, indicating that the spinal cord hemisection was successful. After the rats in the sham operation group were anesthetized, the skin and muscle were sutured after T9–T10 laminectomy, and the spinal cord was not hemisectioned.

### EV intervention

EVs (50 μg in 50 μl PBS) were intravenously injected into rats once a day for a total of three times from the first to the third day after SCI surgery.

### Basso–Beattie–Bresnahan (BBB) scoring

The BBB scores of rats were assessed by two professional researchers who were unaware of the grouping. The total score of BBB used in this study was 21. The rats without any motor function had a BBB score of 0.

### A von Frey filament test

Semmes–Weinstein monofilaments (Stoelting) was used for the von Frey filament test. The fore used in this study was 0.008–300 N. A von Frey filament test was performed 10 min before the SCI modeling, and it was performed on days 1, 4, 7-, 14-, 21- and 28 post SCI.

### Motor evoked potential

The American AXON evoked potential meter was used for the detection of rat motor evoked potential. The stimulating electrode is a self-made pair of needle electrodes, and the distance between the cathode and the anode is ∼0.3 cm. The muscle action potential was recorded on the tibialis anterior muscle of the calf, and the distance between the positive and negative electrodes of the recording electrode was ∼0.5 cm. Another silver needle electrode was pierced under the skin of the tail to ground. The stimulation method was a single direct current pulse stimulation. The stimulation intensity was 10 mA. The frequency was 5 Hz, the wave width was 0.1 ms, and the filter bandpass was 30–1,500 Hz.

### Statistical analysis

The statistical analyses were conducted utilizing one-/two-way analysis of variance (ANOVA), followed by post hoc testing for relevant comparisons. The presented data are expressed as mean ± standard deviation (SD). A significance level of *p* < 0.05 was employed to determine statistical differences. Prior to analysis, the normality of the data was assessed using Anderson–Darling test, D’Agostino and Pearson’s test, Shapiro–Wilk test, and Kolmogorov–Smirnov test. These tests were applied to ensure the assumptions of normality were met before proceeding with the statistical analyses.

## Results

### Identification of BMSCs and BMSC-derived EVs

**Figure 1.**
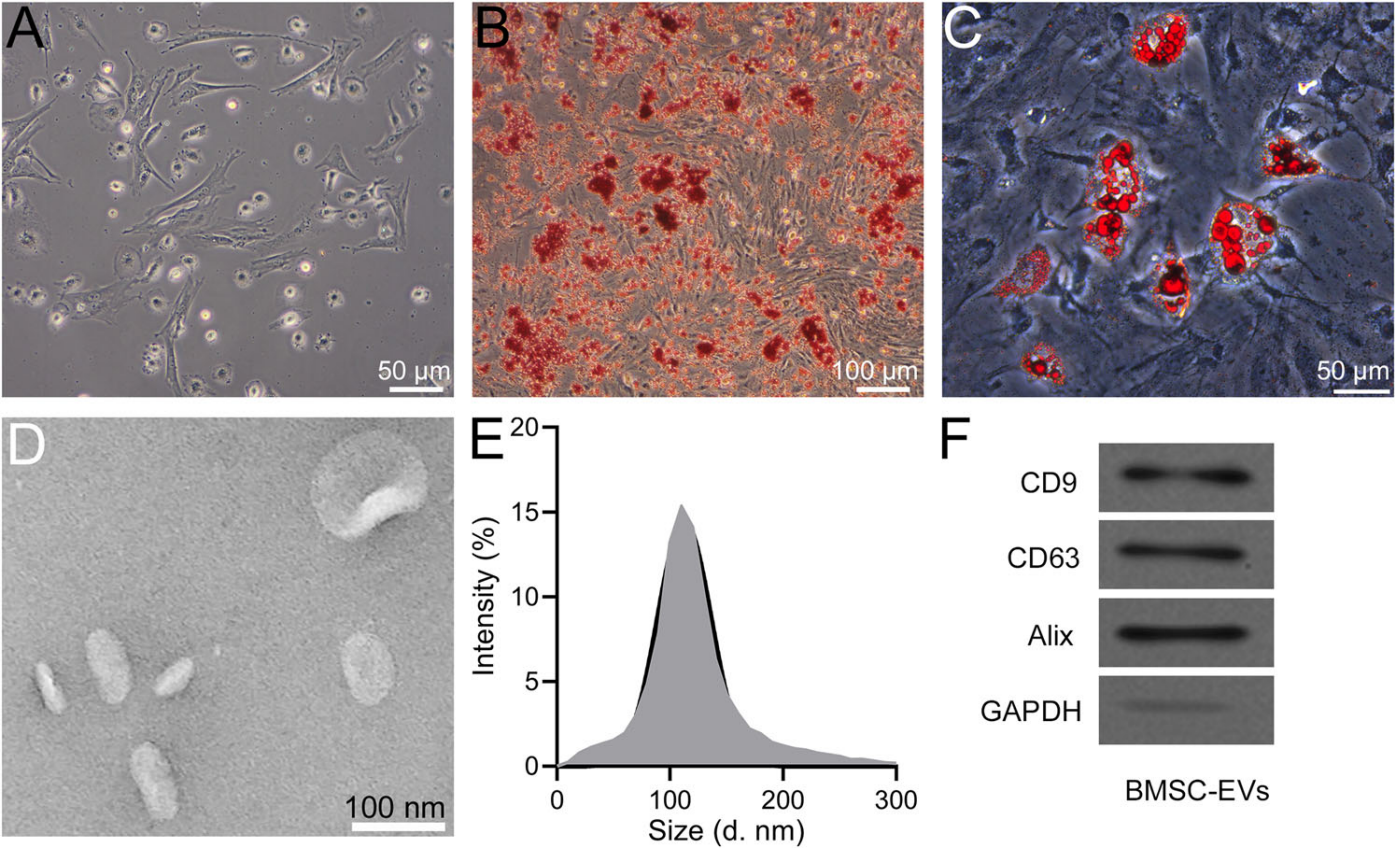
BMSCs and BMSC-derived extracellular vesicles. ***A***, Image of BMSC morphology at passage 3. ***B***, Osteogenic differentiation. BMSCs were stained by alizarin red S to show the matrix mineralization. ***C***, Adipogenesis differentiation. BMSCs were stained with oil red O. ***D***, TEM images of extracellular vesicles. ***E***, Particle sizes of the extracellular vesicle mixture. ***F***, Protein levels of CD9, CD63, and Alix in extracellular vesicles.

To investigate the impact of EVs released from BMSCs, we isolated BMSCs and subjected them to flow cytometry analysis. Cells exhibiting a negative expression for CD34 and CD45, along with positive expression for CD29 and CD105, were identified and characterized as BMSCs (Extended Data Fig. 1). The morphological features of BMSCs are visually represented in Figure 1*A*. Subsequently, the functionality and activity of these BMSCs were further assessed through the induction of osteogenic and adipogenic differentiation in the third generation of BMSCs over a 14 d period. The differentiation potential of BMSCs into osteoblasts and adipocytes was confirmed through alizarin red S and oil red O staining, as depicted in Figure 1*B*,*C*. EVs secreted by the third generation of BMSCs were collected via high-speed centrifugation. Transmission electron microscopy (TEM) images of these EVs are provided in Figure 1*D*. Additionally, Nanoparticle Tracking Analysis was employed to determine the particle diameter of these vesicles, revealing an average diameter of ∼100 nm, as illustrated in Figure 1*E*. The expression of CD9, CD63, and Alix, indicative of exosomal identity, was notably high in these small EVs, as depicted in Figure 1*F*. This thorough characterization underscores the successful isolation and identification of BMSCs, as well as the subsequent collection of small EVs for further investigation.

10.1523/ENEURO.0361-23.2023.f1 Figure S1. Cells negatively expressed CD34 and CD45 and positively expressed CD29 and CD 105 were regarded as BMSCs. Download Figure S1, DOCX file.

### MiR-211-5p targeted COX2 and regulated the expressions of COX2 in BMSCs

**Figure 2.**
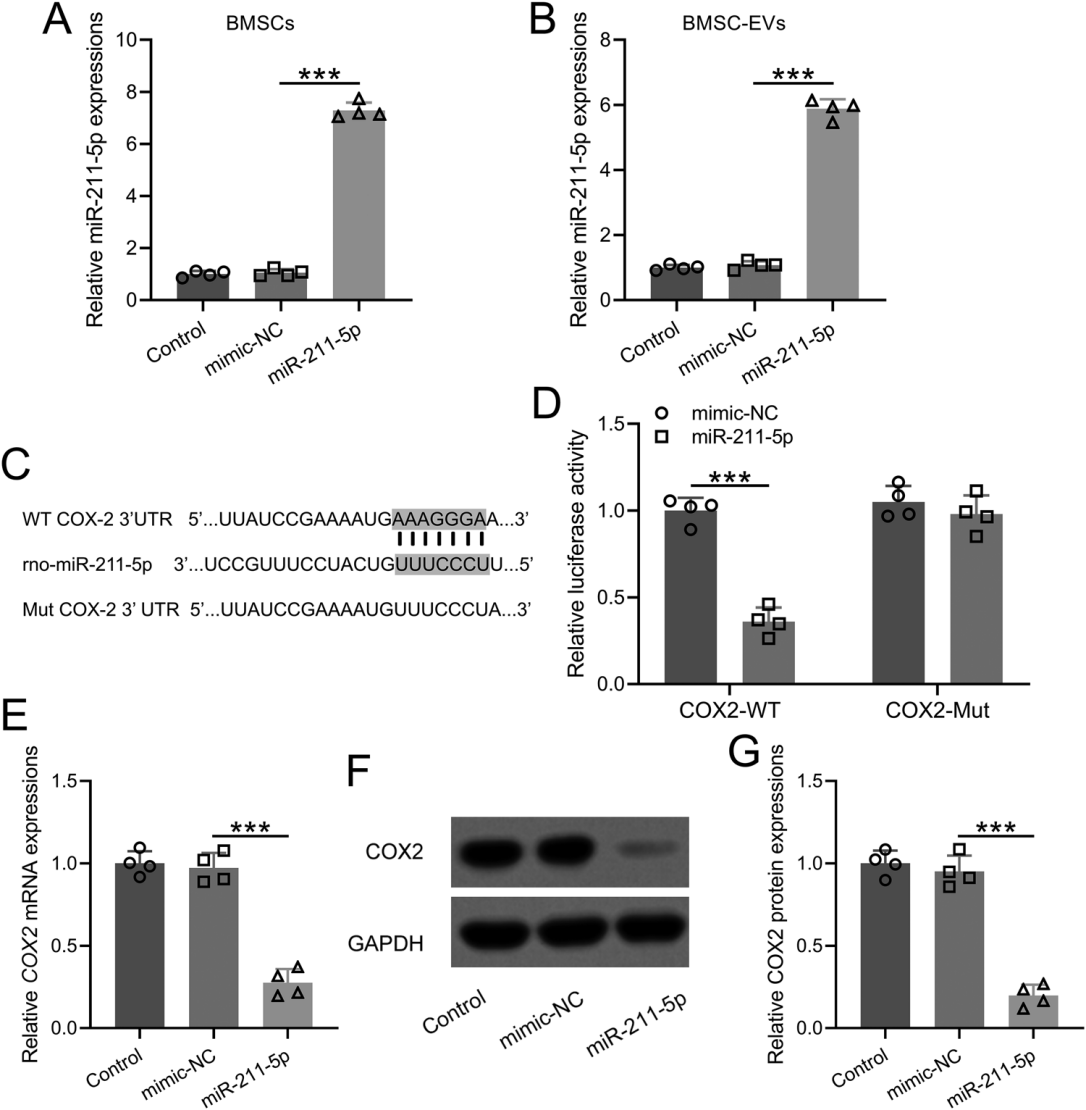
miR-211-5p inhibited the expressions of COX2 in BMSCs. ***A***, The RNA levels of miR-211-5p from BMSCs and miR-211-5p-overexpressed BMSCs. ***B***, The RNA levels of miR-211-5p from BMSCs-derived extracellular vesicles and extracellular vesicles from miR-211-5p-treated BMSCs. ***C***, The suspected binding 3′-UTR region of COX2 mRNA. A mutated 3′-UTR of COX2 is designed. ***D***, Relative luciferase activities. ***E–G***, The mRNA and proteins levels of COX2. *N* = 4. ***p < 0.001. Dunn's and Tukey's post hoc tests.

To investigate the regulatory functions of miR-211-5p on the expression of COX2 in BMSCs, miR-211-5p was transfected into the BMSCs. The expression of miR-211-5p was shown in Figure *2A*. The quantification of miR-211-5p within the small EVs released from BMSCs with miR-211-5p overexpression demonstrated a significant increase compared with the control group, as illustrated in Figure 2*B*. To explore the potential binding sites of miR-211-5p within the 3′-UTR regions of COX2 mRNA, we performed a bioinformatics analysis, and the putative binding sites are presented in Figure 2*C*. Subsequently, luciferase reporter assays were employed using constructs containing the normal 3′-UTR of COX2 (COX2-WT) and a mutated version of the 3′-UTR of COX2. These constructs were treated with miR-211-5p and mimic-negative control (NC). The results, as depicted in Figure 2*D*, indicated that miR-211-5p significantly inhibited the luciferase activity of COX2-WT, while the luciferase activity of the reporter containing the mutated 3′-UTR of COX2 remained unaffected by miR-211-5p treatment. Furthermore, the regulation of miR-211-5p on COX2 was validated at both the RNA and protein levels in BMSCs. Figure 2, *E*, *F*, and *G*, demonstrated that miR-211-5p significantly inhibited both the RNA and protein levels of COX2 in BMSCs, providing conclusive evidence of the suppressive role of miR-211-5p in the expression of COX2 within this cellular context. These findings contribute to a comprehensive understanding of the molecular mechanisms underlying the regulatory functions of miR-211-5p on COX2 in BMSCs.

### EVs released from miR-211-5p-overexpressed BMSCs ameliorated SCI-induced dysfunctions in rats

**Figure 3.**
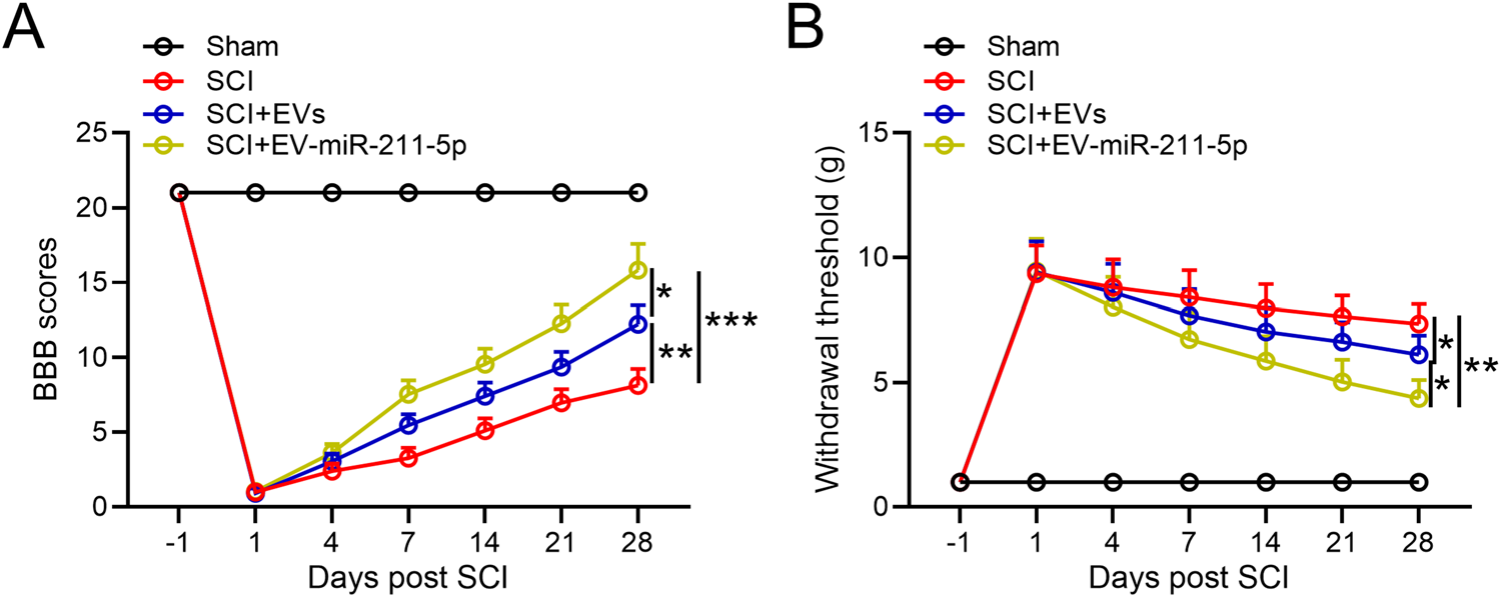
Extracellular vesicles released from miR-211-5p-overexpressed BMSCs ameliorated SCI-induced dysfunctions in rats. ***A***, The BBB scale for rat behavior following surgery. ***B***, The hindpaw withdrawal thresholds in response to mechanical stimulation *N* = 8. Mean ± SD. *p < 0.05, **p < 0.01, ***p < 0.001. Tukey's post hoc test.

To investigate the effects of EVs released from miR-211-5p-overexpressed BMSCs against SCI-induced dysfunctions in rats, were performed the BBB scale. EVs released from normal BMSCs demonstrated an improvement in BBB scores at day 28 post modeling when compared with the SCI group. Notably, the EVs released from miR-211-5p-overexpressed BMSCs exhibited a further significant upregulation of BBB scores in comparison with the SCI + EVs group at the same time point (Fig. 3*A*). Concurrently, hindpaw withdrawal thresholds in response to mechanical stimulation were evaluated at the indicated time points. Figure 3*B* illustrated that the EVs released from miR-211-5p-overexpressed BMSCs significantly lowered the withdrawal threshold in comparison with both the SCI + EVs group and the SCI group at day 28 post modeling. These results collectively suggest a pronounced ameliorative effect of EVs derived from miR-211-5p-overexpressed BMSCs on motor function and mechanical sensitivity in rats with SCI. The observed improvements, as quantified by the BBB scale and hindpaw withdrawal thresholds, underscore the therapeutic potential of miR-211-5p-overexpressed BMSC-derived EVs in mitigating the consequences of SCI.

### EVs released from miR-211-5p-overexpressed BMSCs ameliorated SCI-induced motor evoked potential impairments in rats

**Figure 4.**
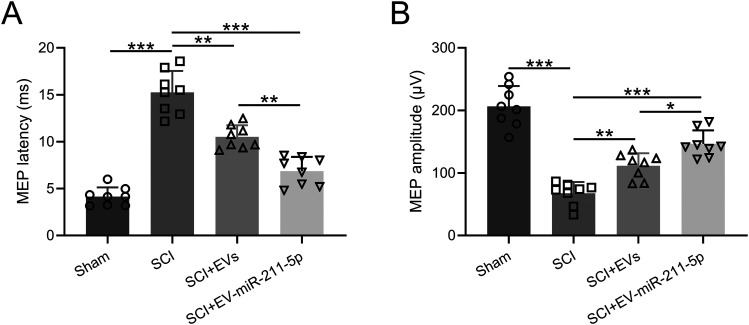
Extracellular vesicles released from miR-211-5p-overexpressed BMSCs ameliorated SCI-induced motor evoked potential impairments in rats. The latency (***A***) and amplitude (***B***) were compared. *N* = 8 for each group. Mean ± SD. *p < 0.05, **p < 0.01, ***p < 0.001. Dunn's post hoc test.

To investigate the protective effects of EVs released from miR-211-5p-overexpressed BMSCs on the neuron function of SCI rats, we detected the motor evoked potential. As depicted in Figure 4*A*, the EVs released from BMSCs overexpressing miR-211-5p exerted a significant downregulation on the latency of motor evoked potentials when compared with both the SCI + EVs group and the SCI group at day 28 post modeling. This observation indicates an accelerated conduction of motor signals in response to the administration of EVs from miR-211-5p-overexpressed BMSCs. Furthermore, Figure 4*B* illustrates that these EVs also elicited a substantial upregulation in the amplitude of motor evoked potentials in comparison with both the SCI + EVs group and the SCI group at day 28 post modeling. This augmentation in amplitude suggests an enhancement in the overall motor response, indicative of a positive impact on the functional recovery of motor pathways. The findings presented in Figure 4*A*,*B* collectively underscore the efficacy of EVs released from miR-211-5p-overexpressed BMSCs in modulating the latency and amplitude of motor evoked potentials, thereby implicating a potential role in ameliorating neural dysfunctions associated with SCI.

### EVs released from miR-211-5p-overexpressed BMSCs ameliorated SCI-induced expressed of COX2 and related inflammatory responses in rats

**Figure 5.**
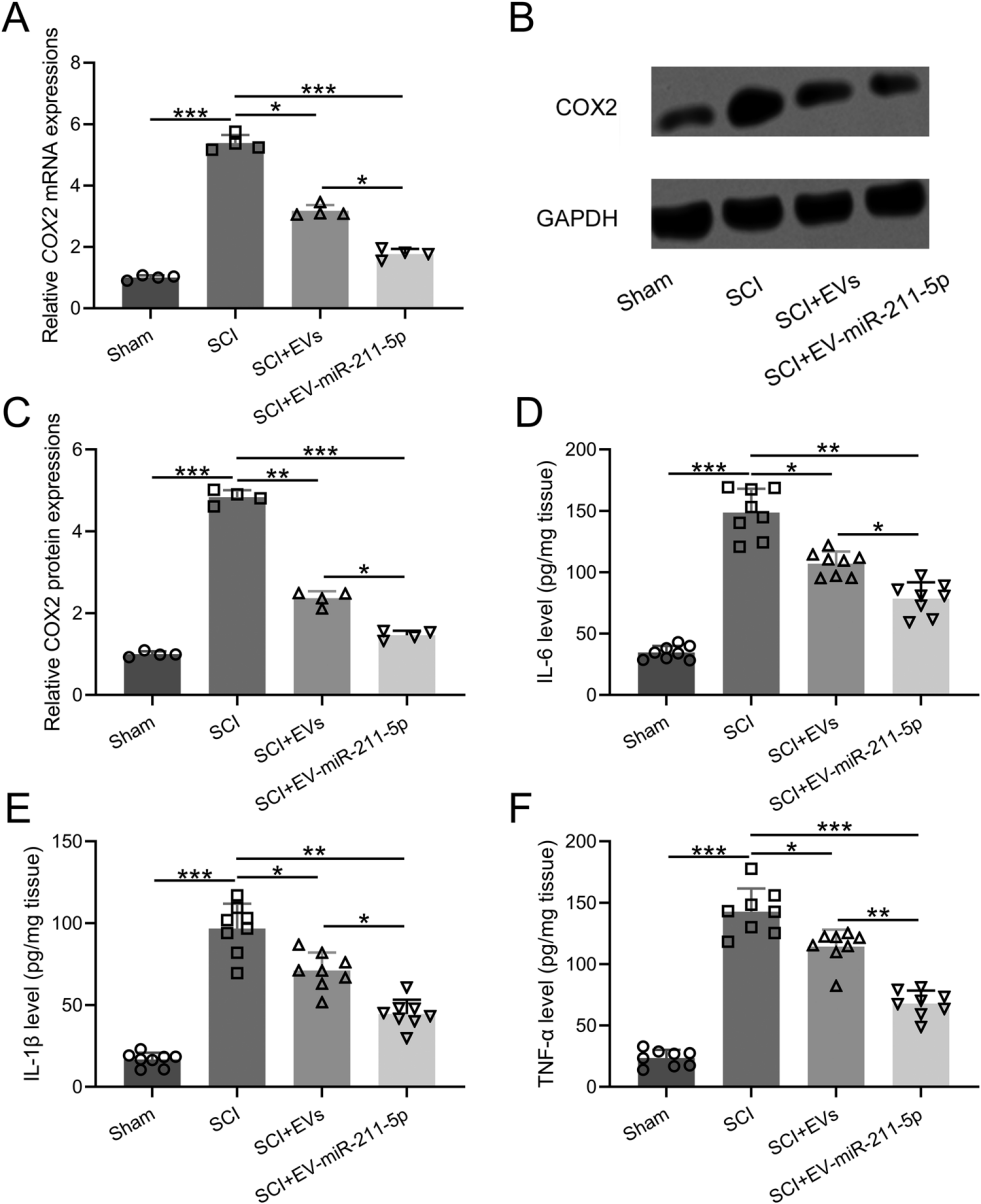
Extracellular vesicles released from miR-211-5p-overexpressed BMSCs ameliorated SCI-induced expressed of COX2 and related inflammatory responses in rats. Spinal cords were collected 28 d after SCI when the behavioral tests were finished. The mRNA and proteins levels of COX2 in spinal cords of rats (***A–C***). *N* = 4 from four repeated experiments (8 rats were used for each group and tissues were mixed). Concentrations of IL-6 (***D***), IL-1β (***E***), and TNF-α (***F***) in spinal cords of rats. *N* = 8 for each group. Mean ± SD. *p < 0.05, **p < 0.01, ***p < 0.001. Dunn's post hoc test.

To explore the protective effects of EVs released from miR-211-5p-overexpressed BMSCs in the SCI rats, we examined the expression of COX2 in the spinal cord of rats. As shown in Figure 5*A–C*, the RNA and protein levels of COX2 were both inhibited in the SCI + EV + miR-211-5p group compared with the SCI group and SCI + EVs group. In addition, treatment of EVs released from miR-211-5p-overexpressed BMSCs significantly downregulated the levels of IL-6 (Fig. 5*D*), IL-1β (Fig. 5*E*), and TNF-α (Fig. 5*F*) in the SCI + EV + miR-211-5p group compared with the SCI group and SCI + EVs group.

## Discussion

A SCI entails significant and profound consequences. Pathophysiological alterations subsequent to such injuries encompass primary and secondary injury phases (Powell and Davidson, 2015; Cowan et al., 2020). Contemporary therapeutic approaches to SCI predominantly focus on alleviating the factors contributing to primary injury and fostering the restoration of damaged nerve tissue. The inhibition of secondary injury and the facilitation of expedited recovery in injured nerve tissue pose formidable challenges in the realm of treatment. Simultaneously, these challenges assume the status of foundational principles within the theoretical framework guiding post-SCI interventions.

In recent years, the previous concept that nerve cells cannot regenerate has become the past with the rise of stem cell engineering and therapy (Nourian et al., 2019). The therapeutic effects of stem cell transplantation on SCI have been widely studied and show a promising prospect (Li et al., 2016). MSCs are a class of pluripotent adult stem cells, which mainly exist in connective tissue and organ stroma (Ding et al., 2011). They have the ability of self-renewal, multidirectional differentiation, and homing. BMSCs have been used to treat various neurological diseases, because of their convenient source, easy culture and amplification, and low immunogenicity (Vilaca-Faria et al., 2019). However, stem cell transplantation has some problems, such as teratogenicity, possible embolism, and immune rejection. It has been found that paracrine function is one of the main mechanisms of stem cell transplantation for the treatment of SCI (Cofano et al., 2019).

EVs are vesicular bodies carrying message factors such as proteins and RNAs (H. Zhang et al., 2020). Many studies have focused on exosomes as transmitters, including long stranded RNA, short stranded RNA, and live double stranded RNA, which are used to transmit signals or kill some specific cells (Xu et al., 2019). The potential regulatory effects of exosomes secreted by mesenchymal stem cells on a variety of life activities of other cells have been reported by many studies (Huang et al., 2020). However, there are few studies to study the therapeutic effect of exosomes secreted by microRNA modified mesenchymal stem cells on various diseases (Cao et al., 2019). Whether the exosomes secreted by the mesenchymal stem cells with overexpression of miRNA and whether the exosomes containing miRNA can regulate the physiological activities of downstream cells are still unclear (L. Zhang et al., 2020).

MicroRNA miR-211-5p is a small noncoding RNA molecule that plays a regulatory role in gene expression. Specifically, miR-211-5p has been implicated in various cellular processes, including but not limited to cell differentiation, proliferation, and apoptosis. Studies have suggested its involvement in the modulation of specific target genes, influencing pathways relevant to physiological and pathological conditions. The interplay between miR-211-5p and exosomes represents a dynamic molecular interaction with implications for cell-to-cell communication and the regulation of cellular processes. In this investigation, we present a pioneering exploration into the potential therapeutic utility of EVs derived from BMSCs overexpressing miR-211-5p in the treatment of SCI in rats. Our findings substantiate that heightened expression of miR-211-5p facilitates the encapsulation of a substantial quantity of miR-211-5p molecules within EVs released by rat BMSCs. Notably, the EVs emanating from these miR-211-5p-overexpressed BMSCs manifest an inhibitory effect on the expression of COX2 in the injured spinal cord of rats. The consequential downregulation of COX2 correlates with a reduction in inflammatory factors within the injured spinal cord.

The observed mitigation of COX2 expression by EVs from miR-211-5p-overexpressed stem cells correlates with an improvement in the recovery of neurological function and motor abilities in rats afflicted with SCI. However, it is imperative to acknowledge the limitations in our research. Specifically, our study exclusively delineates the inhibitory impact of EVs from miR-211-5p-overexpressed BMSCs on COX2 expression within the injured spinal cord. The broader involvement of COX2 in the secondary response to SCI remains unresolved, as does the elucidation of signaling pathways in the injured spinal cord tissue regulated by miR-211-5p via COX2. Consequently, future investigations will incorporate RNA sequencing studies to explicate the role of COX2 in the cascade response triggered by SCI and to identify potential drug targets.

Furthermore, our inquiry reveals a protective effect on nerve tissue in SCI rats through the administration of small EVs rich in miR-211-5p secreted by mesenchymal stem cells. Despite this promising outcome, the translation of this technology to clinical applications encounters formidable challenges, stemming from the intricate nature of exocrine preparation and the ambiguous side effects. Notably, our study omits an assessment of whether the EVs from these mesenchymal stem cells pose any harm to the rats themselves. To address this, future investigations will employ diverse miRNAs in the treatment of mesenchymal stem cells, aiming to discern whether the therapeutic efficacy of these miRNAs in SCI outweighs potential adverse effects stemming from the exosomes secreted by these stem cells in rats.

Moreover, the study delved into the impact of these EVs on the expression of COX2 and associated inflammatory responses within the spinal cord. Our results, as presented in Figure 5, reveal a noteworthy inhibition of both RNA and protein levels of COX2 in the SCI + EV + miR-211-5p group compared with the SCI group and SCI + EVs group. This observation suggests a regulatory role of miR-211-5p in mitigating COX2 expression after SCI. Additionally, the study investigated the effects on proinflammatory cytokines that the treatment with EVs from miR-211-5p-overexpressed BMSCs significantly downregulated the expression of IL-6, IL-1β, and TNF-α in the SCI + EV + miR-211-5p group compared with the SCI group and SCI + EVs group. This indicates a broader anti-inflammatory effect exerted by these EVs.

### Conclusion

In conclusion, we demonstrated that small EVs secreted by mesenchymal stem cells containing a large amount of mir-211-5p had a protective effect on the nerve tissue of rats with SCI in this study. We found that EVs released from miR-211-5p-overexpressed BMSCs ameliorated SCI-induced dysfunctions in rats. EVs released from miR-211-5p-overexpressed BMSCs ameliorated SCI-induced expression of COX2 and related inflammatory responses in rats. Our study can provide potential therapeutic strategies for the treatment of SCI.

## Synthesis

Reviewing Editor: Eran Perlson, Tel Aviv University Decisions are customarily a result of the Reviewing Editor and the peer reviewers coming together and discussing their recommendations until a consensus is reached. When revisions are invited, a fact-based synthesis statement explaining their decision and outlining what is needed to prepare a revision will be listed below. The following reviewer(s) agreed to reveal their identity: Dianna Willis.

In this manuscript, the authors investigate the use of bone mesenchymal stem cell (BMSC) derived exosomes as a therapeutic approach to improving function in a rat spinal cord injury model. They demonstrate that overexpression of miR-211-5p in these BMSC results in reduction in its target mRNA COX2 and improvement in motor behavior following SCI as measured by BBB testing. In addition, mechanical sensory behavior, as measured by von Frey filaments, and motor evoked potentials also significantly improved. Overall, this manuscript focuses on a topic that is likely to be of interest to journal readers, as the therapeutic potential of exosomes and the use of miR-211-5p following CNS injury both show significant promise. Given that, I think there would be interest from a broad spectrum of Journal readers. This is a revised version of a previously submitted manuscript. The authors have addressed most of the primary criticisms of the initial submission, largely focused on a lack of sufficient details in several of the experimental approaches used. As such, the revision is significantly improved. A few remaining issues remain to be addressed, which should result in a publishable manuscript.

1. Some minor issues with langue still persist, which would benefit from an additional round of editing (for example on line 159, what does "pinged" mean?)

2. The authors still do not show the flow cytometry/cell sorting analysis. They discuss it but should consider a figure as well.

3. At what timepoint is the analysis in Fig 5A-5B performed? I am curious if the changes in the levels of the target transcripts is only seen during/immediately after the administration of the miR-211-5p (i.e., sometime during or immediately after the first 3 days following injury) or if it persists until the end of the behavioral experiment (i.e., 28 days). This would provide some insight into the duration of the molecular effects of the early post-injury treatment.
