# Supplementary material for: Small Extracellular Vesicles Released from miR-211-5p-Overexpressed Bone Marrow Mesenchymal Stem Cells Ameliorate Spinal Cord Injuries in Rats
Source: eNeuro. 2024 Feb 9;11(2):ENEURO.0361-23.2023. doi: 10.1523/ENEURO.0361-23.2023 (PMC10866331; doi:10.1523/ENEURO.0361-23.2023)
Supplement: Figure S1. — Cells negatively expressed CD34 and CD45 and positively expressed CD29 and CD 105 were regarded as BMSCs. Download Figure S1, DOCX file. [file eneuro-11-ENEURO.0361-23.2023-s001.docx]

Supplementary materials





Figure S1. Cells negatively expressed CD34 and CD45 and positively expressed CD29 and CD 105 were regarded as BMSCs.
